# 3-D Imaging Systems for Agricultural Applications—A Review

**DOI:** 10.3390/s16050618

**Published:** 2016-04-29

**Authors:** Manuel Vázquez-Arellano, Hans W. Griepentrog, David Reiser, Dimitris S. Paraforos

**Affiliations:** Institute of Agricultural Engineering, University of Hohenheim, Garbenstrasse 9, Stuttgart 70599, Germany; hw.griepentrog@uni-hohenheim.de (H.W.G.); dreiser@uni-hohenheim.de (D.R.); d.paraforos@uni-hohenheim.de (D.S.P.)

**Keywords:** 3-D sensors, optical triangulation, time-of-flight, interferometry, agricultural automation, agricultural robotics

## Abstract

Efficiency increase of resources through automation of agriculture requires more information about the production process, as well as process and machinery status. Sensors are necessary for monitoring the status and condition of production by recognizing the surrounding structures such as objects, field structures, natural or artificial markers, and obstacles. Currently, three dimensional (3-D) sensors are economically affordable and technologically advanced to a great extent, so a breakthrough is already possible if enough research projects are commercialized. The aim of this review paper is to investigate the state-of-the-art of 3-D vision systems in agriculture, and the role and value that only 3-D data can have to provide information about environmental structures based on the recent progress in optical 3-D sensors. The structure of this research consists of an overview of the different optical 3-D vision techniques, based on the basic principles. Afterwards, their application in agriculture are reviewed. The main focus lays on vehicle navigation, and crop and animal husbandry. The depth dimension brought by 3-D sensors provides key information that greatly facilitates the implementation of automation and robotics in agriculture.

## 1. Introduction

Sustainable strategies are in demand in agriculture due to the urgent need to increase resource efficiency in crop production. Agricultural mechanization and intensification have greatly contributed to the development of a food production system able to provide food, feed, fibre and even fuel for the world’s population. Unfortunately, large amount of resources like: fuel, water, herbicides, pesticides, and fertilizers have been intensely employed in this, resulting in a current environmentally unsustainable situation due to the low resource use efficiency [1].

Agricultural automation and robotics can play a significant role in society to meet its future food production needs [2]. These technologies have already lowered production costs, reduced the intensive manual labour, raised the quality of farm products and improved environmental control [3]. One-dimensional (1-D) [4] and two-dimensional (2-D) vision systems have been an integral part of the successful implementation of agricultural automation and robotics in the food production chain. It is believed that the machine vision technology is at an inflection point, moving into a three dimensional (3-D) approach, driven by the improved technology and lower device prices in the consumer market [5]. In the last decade, the number of publications related to agricultural 3-D vision systems has been growing fast. Some reasons contributing to this tendency include the continuous increase in computer processing power, the decrease in cost and size of electronics, the increase in solid state illumination efficiency, the unique non-contact and non-destructive properties of machine vision technology, and the need for greater knowledge and care of the individual crops. The implementation of 3-D imaging systems in agriculture is impeded by the economic justification of using expensive devices for producing relative low-cost seasonal products. However, this may no longer be true since actuators, not vision sensors, are now among the most expensive components in automated and even robotic systems.

**Figure 1:**
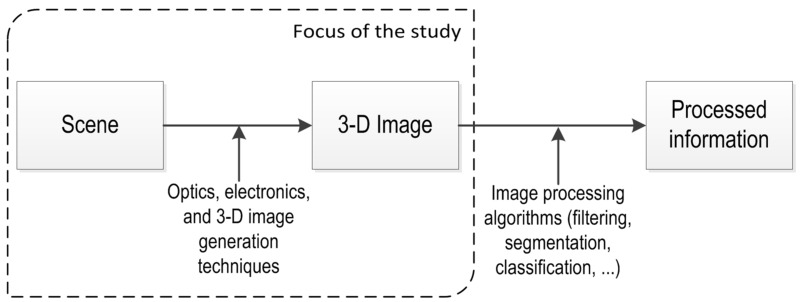
The 3-D image generation techniques are critical for generating robust raw data for useful information extraction.

In 3-D vision systems, a single image of a scene contains huge amounts of information where recovery of depth information is complex. The depth of information is lost in the projection from the 3-D world to the 2-D imaging surface [6]. Due to the extra dimension, a 3-D image increases in the amount of data that needs to be handled, and as a consequence increases the significance of the 3-D image generation techniques. These techniques are vital for handling the extraction of depth information. This is particularly evident in the triangulation techniques where algorithms get a collection of images of a scene and extract the 3-D information out of it [7]. Figure 1 distinguishes between 3-D generation and image processing techniques. 3-D image generation techniques are critical for producing useful 3-D raw data. On the other hand, 3-D image processing techniques are important for processing information out of the 3-D image. They are not considered in the present study since they are highly dependent on the 3-D raw data quality.

Review articles are frequently needed in areas featuring a rapidly growing number of research papers. There are already some reviews detailing the different techniques for 3-D image acquisition [8,9] and even some reviews of 2-D together with some 3-D vision systems in agricultural applications [10,11]. However, there has been no comprehensive review so far that provides an insight into the achievements and potential of 3-D vision systems in agricultural applications.

The aim of this review paper is thus to investigate the state-of-the-art of 3-D vision systems in agriculture, and the role, value, and advantages that only 3-D data can have to provide information about the surrounding structures such as objects, field structures, natural or artificial markers, and obstacles based on the recent progress in optical 3-D sensors. The structure of this paper consists of an overview of the different optical 3-D vision techniques based on the basic principles. Afterwards, their application in agriculture is reviewed. The review specifically focuses on vehicle navigation and crop and animal husbandry.

## 2. 3-D Vision Techniques

A 3-D image is a large collection of distance measurements from a known reference coordinate system to surface points on the objects scene [12]. Depending on the context, a 3-D image is also known as range image, depth map, depth image, or 2.5-D image. In surveying, terms like Digital Terrain Map (DTM), Digital Elevation Model (DEM), and Digital Surface Model (DSM) are commonly used. Several types of spectral waves like light, ultrasound and microwaves can be used to retrieve depth information. 3-D data acquisition with optical techniques is favoured over other alternatives since optical systems allow fast 3-D shape measurement acquisition, high lateral resolution and safety standards compliance [13]. Several classifications have been proposed based on common characteristics, but the one based on the basic principles described by Schwarte *et al.* [14] is widely accepted and provides a detailed insight of the varied possibilities in a well-organized and hierarchical structure. This classification provides more information about the principle behind the construction of a 3-D image, and a more detailed description about the different techniques and applications can be found in the Handbook of computer vision and applications [15]. These basic principles for optical depth measurement are triangulation, time-of-flight (TOF), and interferometry.

### 2.1. Triangulation

**Figure 2:**
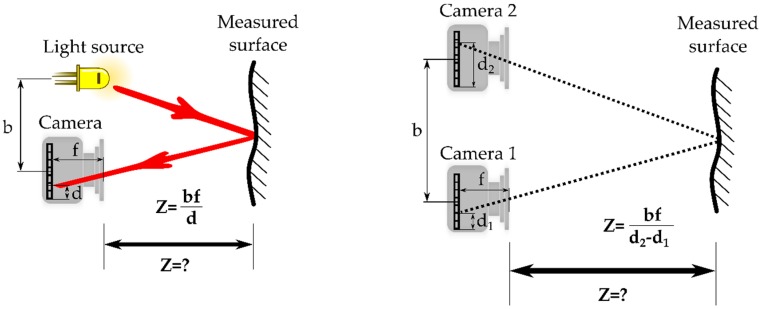
Schematic representation of light beam (**left**) and stereo vision (**right**) triangulation. “**Z**”, depth; “**b**”, baseline length; “**d**”, position of the incoming light beam on the image sensor; and “**f**”, focal length.

Triangulation is a geometrical calculation where the target is one point of a triangle and the other two points are known parts of the measurement system. Measuring the triangle’s angles or baseline, the distance to the target can be determined [16]. Triangulation is the most commonly used principle for depth measurement. Figure 2 shows two typical examples of triangulation based techniques using active and passive illumination.

Triangulation is divided into a variety of techniques based on visual cues to infer depth. Table 1 lists these techniques under the different triangulation approaches. 

There are digital photogrammetry (passive), structured light (active), shading, focus and theodolite measuring techniques. The Xtion Pro sensor (ASUS, Taipei, Taiwan) and the first generation Kinect™ (Kinect v.1, Microsoft Corp., Albuquerque, NM, USA) are examples of consumer triangulation sensors (CTSs) based on the structured light volume technique that use a pseudo random pattern to retrieve depth.

### 2.2. TOF

**Figure 3:**
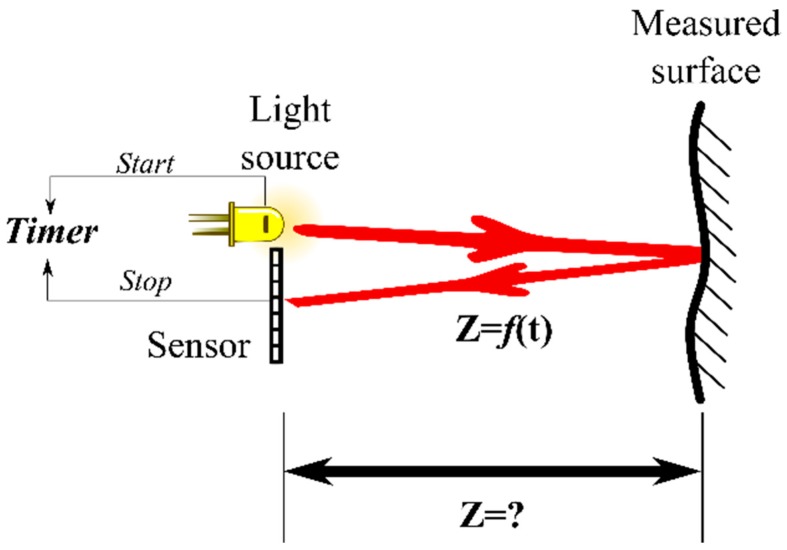
Schematic representation of the basic principle of time-of-flight measurement, where distance “**Z**” is dependent on the time “**t**” that takes a light pulse to travel forth and back.

TOF sensors measure depth using the known speed of light and its time of flight directly or indirectly (Figure 3). Sensors such as LIDARs, Flash LIDARs, 360° 3-D LIDARs, and TOF cameras belong to this category. TOF depth measurement principles can be divided into pulse modulation, continuous wave modulation, and pseudo-noise modulation.

TOF cameras are available since the last decade and are increasingly being used in agricultural applications. They are known in the literature for their low pixel resolution and high cost. However, the second generation Microsoft Kinect™ (Kinect v2), an example of a consumer TOF camera (CTC), has superior technical characteristics at an affordable price. Since there is a patent on this device, the details of its functionality are not openly available; however, Lachat *et al.* [33] assume that the technique behind this CTC is continuous wave modulation TOF.

### 2.3. Interferometry

**Figure 4:**
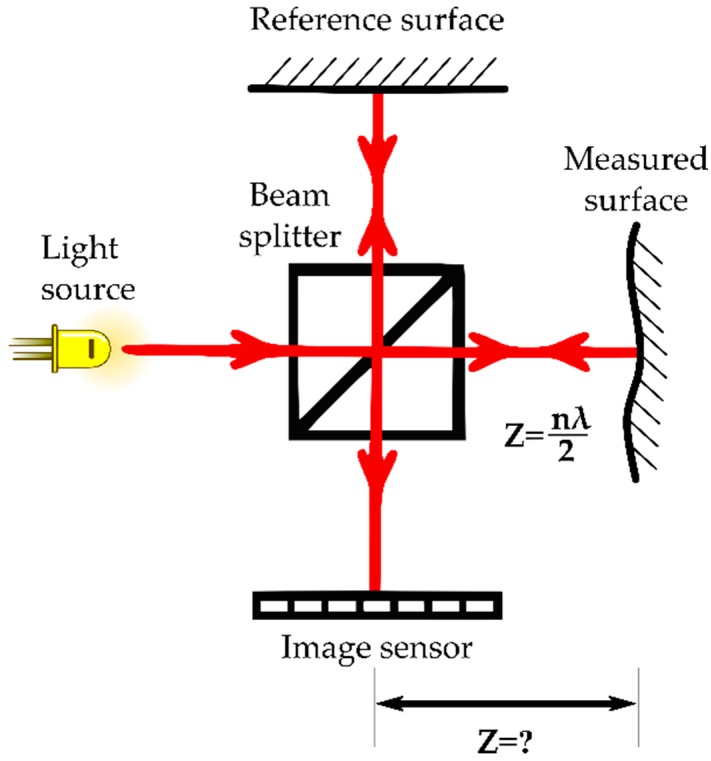
Schematic representation of a Michelson interferometer where the relative depth “**Z**” is directly proportional to the wavelength of the light source “**λ**” and to the number of fringes “***n***”.

Interferometry is the most accurate of the basic principles, with accuracies in the nanometre range. The basic operation of the interferometer consists of splitting a coherent light beam into two, one of which is projected towards a reference mirror while the other is projected towards a sample. Both rays are then reflected back to the beam splitter and projected towards a sensor for integration, where the phase shift between the beams is used to determine the relative depth (Figure 4). Optical Coherence Tomography (OCT) is an interferometric technique able to produce a full tomographic or topographic image depending on the penetration depth of the light. Interferometric techniques are classified into multiwavelength, holographic, speckle interferometry, and white light.

### 2.4. Comparison of the Most Common 3-D Vision Techniques

Since this review paper deals with agriculture, it is important to analyse the most common implementations of the different 3-D vision techniques based on the basic principles. Table 2 presents the advantages and disadvantages of the most common sensor implementation described in the basic principles. The content of Table 2 was formed from an agricultural-based perspective. In the table it is clear that interferometric sensors are very scarce compared to triangulation and TOF ones. Also, the technical characteristics of state-of-the-art sensors are improving the traditional disadvantages of the well-stablished commercial versions. This is particularly noticeable in the modern TOF cameras. CTCs have a good price/performance ratio, thus, they have a lot of potential in agriculture. Smart stereo vision sensors are increasingly common and are able to stream real-time depth measurements.

## 3. Applications in Agriculture

### 3.1. Vehicle Navigation

The agricultural sector is a pioneer in autonomous navigation relying on global navigation satellite system (GNSS). However, GNSS is not available in all agricultural environments at all times. Reactive sensor-based autonomous navigation is based on detailed information regarding the structures surrounding a machine such as objects, field structures, natural or artificial markers, and obstacles. In these type of applications, a superior perception is usually required, and 3-D imaging provides more information about the previously mentioned surrounding structures compared with 2-D. Automated and robotic systems could have faster acceptance by farmers if their safety aspects are well fulfilled [34]. Several reviews [35,36,37] on autonomous navigation of agricultural vehicles have been written, however, there was little focus on the 3-D vision approach.

#### 3.1.1. Triangulation

Autonomous navigation based on stereo vision was successfully achieved in several research studies using different cues. For example, in crop rows, cut-uncut edges, ridges, furrows, artificial markers, swaths and even stubble can be used. Kise *et al.* [38] developed a stereo vision system that uses the 3-D crop row structure for automated guidance; problems like high computational load and blank pixels of some locations (particularly the ones that are further away) were reported, but were addressed by using a reduced resolution frame and filtering, respectively. Rovira-Más *et al.* [39] used the cut-uncut edges of a maize field as a reference for autonomous guidance, they reported that a cloudy sky affected the 3-D point generation and the long maize leaves were blocking the camera, thus, recognizing the importance of the position of the camera. They also faced difficulties related with computational processing, but solved it by reducing the amount of points to a certain range. Hanawa *et al.* [40] used ridges, furrows, and artificial markers for autonomous guidance emphasising the flexibility of their system. They reported limitations when the sunlight was very strong and the projected shadow of the tractor was in the range of the 3-D imaging system. Blas *et al.* [41] developed an autonomous guidance system that uses a swath as the main reference. The 3-D imaging system had problems with the height resolution that failed to detect the difference on a very flat region of the swath. Wang *et al.* [42] relied on stereo vision to track the texture rich surface of a cultivated stubble field and calculate the vehicle’s lateral offset. When the vehicle travelled in straight path, the maximum absolute deviation measured was 50 mm, and although it did not perform well in curved paths, no technical limitations regarding the stereo vision acquisition system were reported. Trinocular vision allows multiple baseline longitudes that complement each other for a more accurate depth measurement at different ranges. Reina and Milella [43] used a trinocular vision and machine learning for ground or non-ground labelling in agricultural environments, reporting a classification precision of 91%. Furthermore, they fused stereo vision with other imaging techniques stating that the combination LIDAR-stereo vision is mutually complementary in many aspects. The classification results were better with the combined sensors than with the single sensors [44].

Tasks such as deformable and rigid obstacle recognition, reliability and operator protection are all considerable with 3-D sensors. Wei *et al.* [45] developed and obstacle detection system using stereo vision to enhance the safety of autonomous vehicles stating the robustness against foreground and background colours, and the limitation regarding the field of view and the number of tracking obstacles. Yang and Noguchi [46] used two omnidirectional cameras to develop a human-detection safety system capable of acquiring a depth image with a reported error of less than half a meter, however, the experiment detects not more than a single human at day time. Nissimov *et al.* [47] developed and obstacle detection system for greenhouse environment using a CTS with only few false positive detections and claiming that it could be used in a computer with limited processing capabilities. Several sources of error were mentioned such as problems with smooth and shiny surfaces, misalignment between the RGB and depth image, time delay (30 s) for a stable depth measurement after a quick rotation, synchronization, and mismatch between the RGB and depth images’ field of view and point of view.

Night-time farming is being investigated in several publications since it provides a convenient environment for image acquisition, and potentially reducing the hazard of an autonomous vehicle colliding with humans. Another advantage is that fruit harvesting robots could become profitable if they are able to provide a 24/7 service. CTCs have a lot of potential for night farming applications since they are able to output an infrared stream. Kaizu and Choi [48] developed an augmented reality 3-D system to assist tractor navigation at night-time. Although it was mainly developed using surveying and blending calibrated video images with computer graphics, it became clear that 3-D sensors could fit well to perform the same task.

#### 3.1.2. TOF

Choi *et al.* [49] developed a navigation system for a combine harvester based on a LIDAR (pulse modulation) mounted on a pan-tilt system that performs a 21° pitching. The system was evaluated under static and dynamic conditions with lateral root mean square (RMS) errors of 0.02 m and 0.07 m, respectively. Yin [50] used a TOF (continuous wave modulation) camera as the main navigation sensor for an agricultural vehicle that targets and follows a human, to further complement the concept of collaborative master-slave and multi-robot systems.

Commercial automatic guidance systems based on 3-D vision already exist in agriculture: CLAAS developed a smart 3-D stereo vision camera called CAM PILOT [51] that tracks different agricultural patterns such as ridges, swaths, crop rows and vineyards using 2-D and 3-D image processing techniques independently or in combination. Also, IFM electronic offers a smart continuous wave modulation TOF 3-D sensor (O3M system) where the emitter is situated in a separate unit from the receiver. It was specifically designed for outdoor use and interferences such as sunlight or materials with different reflective characteristics do not influence the repeatability of the measured data. This systems is able to detect a swath’s contour lines for automatic navigation, and it also provides automatic object recognition of 20 different objects in a range of up to 35 m [52]. Regarding driver assistance systems, CLAAS offers a stereo vision system for automatic trailer fill, called AUTO-FILL [53], for a forage harvester. The camera locates the trailer, tracks the crop jet and hit point, and determines the fill level. An equivalent system called IntelliFill [54] was developed by New Holland (Turin, Italy) but uses instead a TOF camera. Hernandez *et al.* [55] have experimented with a stereo pair from a different perspective using a UAV mounted with a camera system that was previously calibrated, and although the accuracy was not outstanding, the processing speed for updating the results was appealing, however, issues like the influence of wind in the stability of the UAV in the open field and thus the quality of the images (blur) still need to be evaluated. Finally, Naio Technologies (Ramonville-Saint-Agne, France) has developed a commercial field robot [56] for mechanical weeding that relies on stereo vision for autonomous navigation between the crop rows. Initially, they relied on a light sheet LIDAR (pulse modulation) for navigation within the crop rows, then upgraded the vision system to stereo vision, claiming that with it, they have a more accurate positioning and behaviour of the field robot and are able to detect smaller plants.

### 3.2. Crop Husbandry

Important parameters like crop growth status, biomass estimation, height, shape, nutrient supply, and health status are better analysed using 3-D sensors since the acquired data can be used for measuring or correlating the previously mentioned crop parameters. If the data is geo-referenced, individual crop plant treatment can be applied.

Recently, Li *et al.* [57] reviewed the state-of-the-art in plant phenotyping, where different 3-D vision techniques are used. It was concluded that their refinement and development will accelerate the phenotyping process. Rosell and Sanz [58] reviewed the geometric characterization of tree crops, and Wulder *et al.* [59] of forest trees where airborne LIDAR has become an important tool for characterization. Vos *et al.* [60] reviewed plant modelling (virtual plants) which becomes increasingly important for conducting virtual experiments that otherwise would take years to perform in field conditions. This is closely related with the quantification of plant properties.

A number of reviews related with quality inspection and grading of agricultural products were conducted. Moreda *et al.* [61] contributed with a review on the different vision technologies for size determination and grading. They expect 2-D imaging to be increasingly substituted by 3-D, and consider 3-D multispectral scanning (combination of multispectral data with 3-D surface reconstruction) a promising technology. Bac *et al.* [62] reviewed harvesting robots for high-value crops where commercial versions are already available for strawberry harvesting, but the price/performance ratio still needs to improve in order to gain acceptance by farmers. A recently concluded research project (CROPS [63]) showed the recurrence in the use of 3-D sensors in harvesting robots.

#### 3.2.1. Triangulation

Triangulation based techniques using UAVs have been thoroughly investigated. Since these aerial vehicles are very cost effective compared to airplanes, and the images are of higher resolution compared with satellites, more research and commercial applications can be foreseen. Structure from motion (SfM) has a big potential in aerial applications involving UAVs, which will be increasingly integrated in future agricultural practices, replacing solutions like satellite or manned aircraft. A number of open source software for 3-D reconstruction are available such as 123D, ARC3D, Photosynth, Visual SFM, Bundler + PMVS2 and MicMac. Recently, Jay *et al.* [64] developed an automatic platform for open-field phenotyping using MicMac. They found that SfM is a convenient technique since intrinsic camera parameters are automatically estimated, therefore, camera calibration is not required. However, they encountered problems like occlusion and plant changing position from one image to the other due to the wind. Santos *et al.* [65] combined SfM with multi-view stereo to produce dense point clouds of a basil specimen (*Ocimum bacilicum*) and an ixora specimen (*Ixora coccinea*) for indoor plant phenotyping*,* however, they stated that the method is limited to not too dense plant canopies due to occlusion and matching problems, aside from the time-consuming image acquisition. The generation of DEMs by means of SfM is an increasingly common practice, and yet it remains unexplored, where useful information can be obtained for soil erosion, hydrological phenomena, and gullies monitoring [66]. Zarco-Tejada *et al.* [67] used and off-the-shelf colour camera, without the infrared filter, mounted on a UAV to acquire high resolution (5 cm·pixel^−1^) DSMs for canopy height quantification. They obtained R^2^ determination coefficients of up to 0.8 compared with the manual measurements, proving that this inexpensive method can provide accuracies as good as the more costly airborne LIDAR. The potential disadvantage of the methodology is the high image overlapping and the low altitude requirements of UAVs flights. Geipel *et al.* [68] acquired aerial images from a UAV and combined 3-D shape information with the RGB spectral information for estimating corn grain yield. They obtained R^2^ determination coefficients of up to 0.74 using three different linear regression models, stating that dense point cloud generation requires high computational power, therefore, they downscaled the images by a factor of two.

Automated crop yield estimation, particularly in orchards and open field scenarios, is of great interest because is a very important parameter for farm management. It is a time consuming and labour intensive activity suitable for automation. Herrero-Huerta *et al.* [69] proposed an automatic process for ground vineyard yield estimation by acquiring five images with an off-the-shelf camera and reconstructing the grape clusters (using SfM technique) at a close range, reporting that the main constrains depend on weather conditions and suggesting the use of artificial light and light diffusers to overcome them. Moonrinta *et al.* [70] also proposed a method for pineapple plantations, considering SfM a promising technique, but recognizing that more work needs to be done to increase the accuracy of their recognition and tracking pipeline. Wang *et al.* [71] used an autonomous stereo vision system for yield estimation of an apple orchard that works at night using ring flashes to illuminate the scene, they reported problems due to occlusion, specular reflections, colour heterogeneity, and a bias in the shape-from-stereo algorithm that caused the apple location to be estimated closer to the camera. To solve the bias problem, they placed artificial landmarks every three trees to recalibrate the vision system.

3-D models of trees, plant and agricultural structures are on demand as they help substitute difficult and expensive experiments. Model-based design reduces costs by avoiding redesign and removing the necessity to build a real prototype for experiment and evaluation [72]. Virtual simulation requires 3-D information of agricultural structures to create a model based on real information in order to pave the way for the following robotic application like harvesting, thinning, pruning, *etc.* Plant phenotyping is very important for plant breading, not just for increasing productivity, but also for minimising the effects of global warming in future farming. Table 3 shows that most of the autonomous phenotyping platforms are for research purposes and rely on 3-D vision (mainly triangulation). It can be seen that shadowing devices are commonly used to maintain constant lighting conditions. The ones that do not have, it is because they are in indoor or greenhouse environments where light can be easily controlled.

Shape-from-silhouette (SfS) has proved to be effective in characterizing the often complex plant architecture. Noordam *et al.* [87] evaluated several 3-D image acquisition techniques and selected the reverse volumetric intersection technique, which is related to SfS, to obtain the best model of a rose with overlapping leaves for the 3-D vision system of a robotic rose cutter. They found this technique attractive because the addition of more cameras from different angles resulted in more information, and to a lesser extent in more processing (if multiple cameras are considered). The task of rose cutting consists in locating the stem and trace it down until the cutting position by taking multiple images to generate the depth image [88]. Tabb [89] tried to reconstruct trees based on the SfS technique taking into consideration that the trees do not contain concavities, but some noisy regions where present and post-processing filtering was required. Billiot *et al.* [90] evaluated Shape-from-focus technique using a monocular camera and two power LEDs. They also developed an acquisition platform that performs a controlled displacement perpendicular to the ground to acquire a stack of 3-D images of wheat ears. The focus value of each pixel in every 2-D image was used to obtain the depth information. They considered that the indoor system needs further development, but it can be translated to open field applications like crop characterization and yield estimation.

Jin and Tang [91] developed an interplant spacing measuring system for corn plants at an early stage, using stereo vision. The system is able to detect almost 96% of the corn plants but with less accuracy at detecting the centre position (62% to 74%) with a processing time between 5 and 20 s. Zhao *et al.* [92] used a light beam triangulation sensor mounted on a 2-D scanning platform to obtain the 3-D shape of zucchini (*Cucurbita pepo*) leaves, for detecting the water stress of the plant by tracing its morphological traits (wilting). They consider that little research has been done using a 3-D approach, even though it provides more reliable information about the wilting behaviour in response to water stress. Piron *et al.* [93] used structured light volume sequentially coded technique to discriminate between weed and crop plants at an early stage by using the difference in height as the main cue. They reported several problems such as limited projector depth of field, high dynamic range scene, internal reflections, thin objects and occlusions. Additionally, they also reported the solutions for every problem. Lino *et al.* [94] used structured light volume shadow Moiré to reconstruct the 3-D shape of pears. The results were compared with the more precise light sheet triangulation technique to evaluate its accuracy by correlating the depth measurements with R^2^ determination coefficient between 0.93 and 0.99, they reported visual noise in a small region but failed to explain the reason of it.

Šeatović *et al.* [95] used a light-sheet triangulation sensor (smart 3-D camera) for the development of a real-time broad-leafed weed (*Rumex obtusifolius*) detection and herbicide spraying prototype in grasslands. The detection rate was high, but decreased when clover or other broad-leafed plants were present. They concluded that a 3-D approach offers by far a more robust segmentation (with the help of height information) and classification of the leaves compared with a 2-D. Wolff [96] developed an open field light-sheet triangulation system for plant phenotyping that consists of two cameras to reduce occlusion, arranged in an enclosed platform. The system scans around 2200 plants per working day, but requires two operators for moving the platform between each acquisition process. The combination of 3-D shape and spectral information are useful for farm management, and they can be acquired either from the ground [97] or from the air [98]. Strothmann *et al.* [99] fused three light sheets (405, 532, and 650 nm) in a triangulation system using a single camera allowing not just 3-D shape reconstruction, but also to gain reflectance information.

Innovative applications have been developed with stereo vision like the inside tyre-terrain contact profile measurement of an off-road vehicle [100], which can be potentially useful for agricultural machinery testing and soil compaction analysis. The authors mention the complexity of the preparatory steps before depth calculation such as camera calibration, stereo rectification, correspondence problem, 3-D point computation and point cloud scaling. Shape-from-shading technique has been used to improve a common problem of 2-D vision systems for apple quality grading, where the stem-end or calyx could be incorrectly classified as a defect. Jiang *et al.* [101] compared a traditional 2-D detection approach with a 3-D vision system based on Shape-from-shading. The result was a decrease of 30% in the overall error rate by using the 3-D approach, however, a zigzag effect at the apple’s boundary was generated (in interlaced video) due to the high speed of the apples on the conveyor.

**Figure 5:**
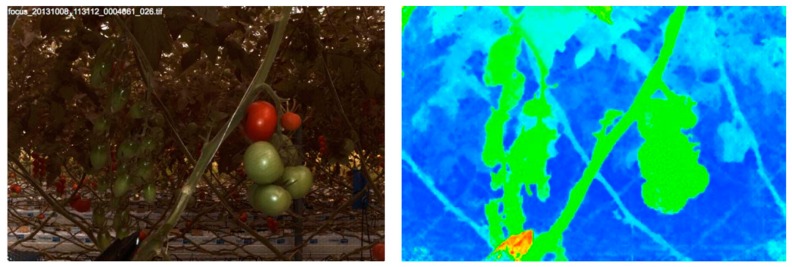
RGB (**left**) and depth image (**right**) using a light field camera (reproduced from Polder and Hofstee [103]).

Advances in technology have allowed the creation of new devices inspired from old theoretical concepts, which is the case of the development of commercial light field cameras. They provide 4-D light field information, allowing 3-D reconstruction, and were evaluated by Möller *et al.* [102] for cereal phenotyping in open field using a multi-sensor platform (BreedVision [75]). Polder and Hofstee [103] also evaluated the same light field camera (Raytrix GmbH, Kiel, Germany) for tomato plant phenotyping in a greenhouse (see Figure 5), stating several disadvantages such as computationally (depth image calculation) and economically expensive, one aperture setting, and limited field of view. Vision Robotics [104] developed a robotic pruning prototype for grapevines based on stereo vision. The enclosed system was designed to control the lighting and protect the two robotic hands with cutter end-effectors, but no information is available regarding the technical limitations of the system.

Although not many effective technologies are available today, soil sensing is of high importance. The vertical dimension of soil properties is of high interest and hence, 2-D sensors can only provide information about structures at the field surface. For tillage operations, it is increasingly important to know about soil roughness and 3-D vision sensors provide a fast acquisition solution. Interestingly, under ideal light conditions Marinello *et al.* [105] successfully measured the soil roughness using a CTS, under favourable lightning conditions, highlighting the effects of oversaturation (due to excessive sunlight exposure) as the main limitation.

#### 3.2.2. TOF

With regard to the use of scout robots, Garrido *et al.* [106] reconstructed maize plants by overlapping LIDAR point clouds using a field robot for data acquisition and a robotic total station for geo-referencing the point clouds, relying on sensor fusion, filtering and processing to reconstruct the 3-D plant structure, and concluding that the orientation of the 3-D sensor is very important. Weiss *et al.* [107] used a low pixel resolution 3-D LIDAR (pulse modulation) to evaluate different machine learning classifiers in an indoor environment. They achieved a classification precision of nearly 99% with one of the trained classifiers (simple logistic regression) using plants of six different species. Afterwards, they used the same 3-D LIDAR for plant detection and mapping in outdoor conditions with a plant detection rate of about 60%. Even though the plant detection rate in the open field was not outstanding, and the sensor’s pixel resolution was poor (29 × 59 pixels), the authors emphasised the advantages of the sensor (reliability under different light and weather conditions) and considered it as the most promising sensor technology for agricultural robotics [108].

Saeys *et al.* [109] evaluated two light-sheet LIDARs, a continuous wave and a pulse modulated, to estimate wheat ear density and crop volume mounted on a combine harvester. They successfully predicted the crop density by conducting experiments with different crop densities (controlled), speeds and vibrations. The LIDAR hits were used to reconstruct the 3-D field in post processing but better results were obtained using the continuous wave LIDAR since its scanning rate were intrinsically higher than the pulse LIDAR.

Nakarmi and Tang [110] developed a system for sensing inter-plant spacing, using a state-of-the-art TOF camera (continuous wave modulation), that was fully covered to protect it from direct sunlight and wind. They mention the superiority of TOF cameras compared to a conventional stereo vision sensor, but also their limitations like the small field of view and the low pixel resolution. Adhikari and Karkee [111] developed a 3-D imaging system for automatic pruning to identify unwanted branches and locate pruning points in a trained apple orchard. 90% of the pruning points were correctly located using a TOF camera (continuous wave modulation).

Gongal *et al.* [112] investigated the fusion of a 2-D camera with a TOF camera (continuous wave modulation) for apple yield estimation in trained orchards. They were able to recognize 88% of the apples emphasizing the significant increase of visibility when the images are captured from both sides of the tree canopies, rather than just from one side. But they acknowledged that the major challenge was the limited visibility of apples, where some of them were completely occluded by leaves and branches. Tanaka and Kataoka [113] used a light-beam triangulation scanner to acquire the 3-D shape of a rotary tiller blade to investigate if a 3-D printed replica made of resin could perform similarly for low-cost prototyping in the effort of improving the design/performance of the rotary tiller blade.

**Figure 6:**
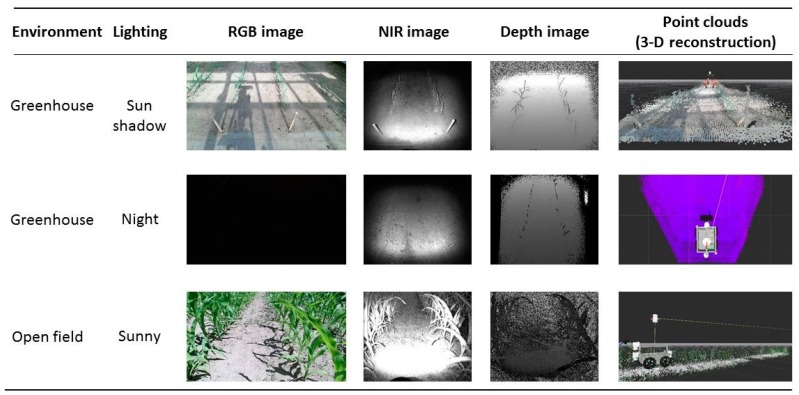
Reconstruction of maize plants using a CTC mounted on a field robot in different agricultural environments (reproduced from [114]).

Vázquez-Arellano *et al.* [114] have emphasised the flexibility and possibilities of CTCs in agricultural applications for different agricultural environments and conditions, since they were able to reconstruct and geo-reference maize plants in a greenhouse and open field environments under different lighting conditions (Figure 6). Deepfield Robotics is also relying on a CTC for plant phenotyping in their Bonirob field robot [74], but for acquiring stable images, they rely on a shadowing device that houses the CTC together with an artificial light source.

#### 3.2.3. Interferometry

**Figure 7:**
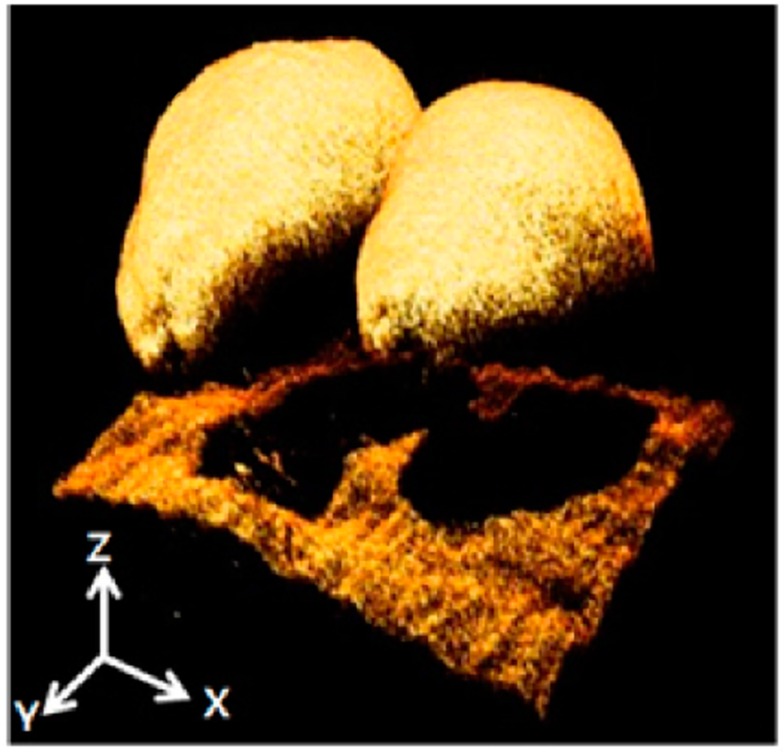
3-D reconstruction of melon seeds based on interferometry (reproduced from [115]).

Interferometry has been used for a long time to measure plant growth or motion changes under different stimuli. Currently, interferometric techniques are being investigated using a 3-D approach for seed inspection and quality control. There is a worldwide rush for preserving genetic pool of different crops in seed banks for future breeding requirements. Lee *et al.* [115] used optical coherence tomography (OCT) based on white-light interferometry to detect infected melon seeds, until now, it is one of the few examples of 3-D reconstruction using interferometry in agriculture (Figure 7). 

Later on, they did the same for cucumber seeds [116]. Barbosa and Lino [117] used electronic speckle interferometry (ESPI) for 3-D shape measurement of a peach concluding that the technique is promising for quality control of agricultural products with smooth and delicate tissue. Madjarova *et al.* [118] also used ESPI for flower blooming growth analysis that can provide useful information of the effects changing weather patterns in flowering (which is sensitive to temperature variability) and thus crop production. This study shows that a high resolution camera is important to resolve high fringe densities. Plant movement analysis is other application area, where Fox *et al.* [119] relied on holographic interferometry to measure motion changes undergone by a mature *Stapelia variegate* under phototropic stimuli, where a reference object was used to detect unwanted movements. In the other hand, Thilakarathne *et al.* [120] relied on white-light interferometry to measure the nanometric intrinsic fluctuations of rice (*Oriza sativa L*.) exposed to different ozone concentrations to investigate the damage and recovery of the plant. They explained that the usage of interferometry was limited because of two main factors: complexity of implementation and the optical properties of the plant itself (highly scattering surface).

### 3.3. Animal Husbandry

#### 3.3.1. Triangulation

Milking robots have been in operation for the last two decades maintaining a steady growth and until recently, they had been the only commercial robots available in agriculture. Current milking robots use light sheet triangulation to estimate the position of the teats, but some experiments have been evaluating alternatives. Ben Azouz *et al.* [121] evaluated a stereo vision system together with thermal infrared acknowledging the difficulty of obtaining a robust disparity estimation particularly in areas of homogeneous colour or occlusion. Similarly, Akhloufi [122] compared two TOF cameras and a CTS, with the latter giving the overall best results mainly because of its superior pixel resolution and colour output.

Early attempts to reconstruct a pig’s surface using light-volume triangulation were done by Van der Stuyft *et al.* [123] without achieving good resolution. They reported the potential of the technique, but also the necessity to have expert knowledge of vision algorithm development and hardware implementation. Ju *et al.* [124] used high resolution cameras (4500 × 3000 pixel) to achieve pig surface resolutions of approximately 0.4 mm pixel^−1^ based on multi-view stereo, but difficulties arose with regard to the discrimination between foreground (pig) and background surfaces, as well as residual corrupted range measurements due to pig’s background occlusion. Automation in slaughtering facilities has allowed a surge in the use of structured light techniques for meat cutting, grading, sorting, and yield calculation since this activity is mainly done in indoor environments where illumination conditions are controlled [125].

Cattle monitoring was discussed by Viazzi *et al.* [126], who compared a 2-D camera with a CTS for lameness detection concluding that the 3-D approach overcomes the limitations of the 2-D, however, CTSs have also limitations such as their sensitivity to sunlight and small field of view. Kawasue *et al.* [127] used three CTS for evaluating the quality of cattle with accuracies of up to 93% compared with manual measurement. An important source of error was caused by the body hair of the cattle. Similarly, Kuzuhara *et al.* [128] used a CTS to estimate parameters like biomass and milk yield of Holstein cows. They obtained R^2^ coefficient of determination of 0.8 for body weight and 0.62 for milk yield. They mention limitations regarding the sensitivity of the sensor to natural light, but this problem was solved by performing the experiments inside the cowshed.

Animal welfare, health monitoring, indoor navigation of robots, feeding and cleaning robots are some examples of the many tasks where 3-D sensors could be included in animal husbandry. Several 3-D imaging techniques can be used since most of the time the measurements can be done in an indoor environment. Animal husbandry has experienced recent advances in quality evaluation and monitoring. There is a need to develop integrated monitoring systems that can measure important performance parameters including physiological variables like shape, size and weight. Menesatti *et al.* [129] developed a low-cost stereo pair system for measuring such parameters for sheep with webcams, reporting R^2^ determination coefficient of 0.8. Pallottino *et al.* [130] used a similar system for measuring body traits for breeding Lipizzan horses, reporting a high correlation coefficient (*r* = 0.998) between manual measurements and stereo vision. Wu *et al.* [131] developed a system for swine monitoring, using three high-resolution stereo vision systems (side, rear and top) for further integration and overall 3-D shape reconstruction. Aquaculture production currently supplies nearly 50% of the fish consumed in the world and the farmers in this sector face major difficulties in monitoring their fish stock. According to a review by Zion [132], several stereo vision systems have been used for measuring individual fish dimensions and mass. Their further improvement could lead to the development of a system capable of estimating the overall biomass and monitoring of fish welfare. However, problems like fish occlusion and poor water transparency need to be addressed.

#### 3.3.2. TOF

Research has been conducted on the development of a robot for herding milking cows [133]. It uses a 360° 3-D LIDAR as its main navigation sensor. Several aspects like response of the cows to the presence of a robot, remote controlled operation, and software algorithms for detecting and tracking were assessed. After three herding tests, the authors showed that remote herding was possible with the potential to improve animal welfare.

### 3.4. Summary

Table 4 lists the technical difficulties so far encountered in the reviewed papers, with the techniques based on the basic principles in agricultural applications. The table shows many comments on technical difficulties regarding stereo vision, and that is expected since it is economically affordable and has been fully studied compared with other techniques. Also, a common technical difficulty is the sensitivity against natural light, where shadowing devices are commonly used to maintain the illumination condition as constant as possible. Occlusion is also a very recurrent problem, here, 3-D sensor pose and position during data acquisition play an important role to minimize occlusions.

## 4. Discussion

New commercial 3-D capable optical sensors such as light field and polarization cameras have recently appeared. The latter provide information about the polarization properties of the image. This information is used by the Shape-from-polarization technique for 3-D shape reconstruction. The previously mentioned sensors rely on triangulation techniques that were not included in the classification proposed by Schwarte *et al.* [14]. The Austrian Institute of Technology [134] has recently developed a dynamic stereo vision camera that continuously rotates to generate a real-time 360° 3-D view. This camera exploits the high sampling rate and low latency capabilities of the dynamic vision sensor (DVS) that only senses changes at a pixel-level, caused by movement, significantly reducing the amount of acquired data. Parallel computation, accelerated by the incorporation of field programmable gate arrays (FPGAs), has enabled the emergence of 3-D smart cameras that have embedded processing. In the case of triangulation based sensors, the depth map can be generated as an output stream, thus, real-time measurements can be performed. Velodyne [135] has released a 360° 3-D LIDAR that is relatively cost-effective and compact in size. Since this type of sensor is preferred in military and automotive autonomous applications, its presence could be also expected in future agricultural scenarios.

Several trends have been detected during this extensive literature review. Any 3-D vision sensor has its disadvantages and advantages, therefore, complementary 3-D sensor fusion provides a more robust performance depending on the application. Also, there is no 3-D sensor completely immune to noise sources such as natural light, sunlight intensity variations, adverse weather conditions (rain, snow, mist and dust), and light reflectivity differences (due to colour and texture). The outdoor range and resolution of TOF cameras are both expected to increase, while the cost per pixel decreases [136]. Several TOF cameras have been tested and compared for agricultural applications by Kazmi *et al.* [137] and Klose *et al.* [138], and although there are still several noise sources that affect them, the technical characteristics of some versions are outstanding and efforts to extend the range of TOF cameras are taking place. Higher resolution TOF cameras (pulse modulation) are already commercially available and reaching resolutions of 4.2 Megapixels [139]. Also, higher measurement ranges are commercially available like the previously mentioned smart TOF 3-D sensor (continuous wave modulation) by IFM electronics that has a measuring range of up to 35 m. Consumer 3-D sensors have had a big impact in agricultural automation and robotics research even with their outdoor limitations, where shading devices have so far minimized the problem.

## 5. Conclusions

Currently, 3-D sensors are becoming smaller, smarter, and cheaper. Therefore, technology breakthroughs are already possible if enough research were commercialized, a statement justified by the fact that some commercial implementations in agriculture are mentioned in this paper. Since agricultural environments are substantially complex, 3-D vision can play an increasing role in enhanced perception that could be suitable in a number of applications in every agricultural scenarios. The true value of 3-D data lies precisely in the superior sensing capabilities compared to 2-D. Several market forecasts have recently appeared around topics such as LED lighting, 3-D sensors, UAVs, and agricultural automation and robotics. Almost all of them forecast a profitable market for these interconnected topics by the end of the decade. Some 3-D techniques have either not been tested (*i.e.*, photometric stereo, shape-from-polarization, shape-from-zooming, flash LIDAR) or more research has to be done with the rich variety of 3-D imaging techniques in agricultural applications (*i.e.*, interferometry, light field, CTCs). 2-D and 3-D fusion is very promising, since it takes the advantages of the two, and has proved to be useful for either obtaining more information about the object’s surface or facilitating the image segmentation process. All 3-D sensors are sensitive in one way or another to sunlight, however, more research needs to be done to reduce its effects and stop relying on shading devices. As a matter of fact, a positive effect of this disadvantage is that autonomous night farming could be investigated more thoroughly since 3-D sensors behave properly in this environment.

This review presents the rich variety of 3-D imaging techniques that have not been tested, the potential of the ones that have already been tested (due to the increase of their technical characteristics), the working principle behind the 3-D imaging sensors, and the potential of consumer 3-D sensors in agricultural applications. The pace of change in 3-D imaging technology is accelerating, therefore, the possibilities of this technology are immense. Reasons like reduced labour availability, scarcity of natural resources, and consumer demand for quality products are driving the need for automation in agriculture. Since 3-D vision is a key technology for automation, more implementations are yet to come.

## Figures and Tables

**Table 1:** List of some triangulation techniques for 3-D image generation found in the literature for different visual cues.

Triangulation Approach	Visual Cue	3-D Image Generation Techniques
Digital photogrammetry	Stereopsis	Stereo vision [17]
		Multi-view stereo [18]
		Multiple-baseline stereo [19]
	Motion	Structure-from-motion [20]
		Shape-from-zooming [21]
		Optical flow [22]
	Silhouette	Shape-from-silhouette [23]
		Shape-from-photoconsistency [24]
		Shape-from-shadow [25]
Structured light	Texture	Shape-from-texture [26] Shape-from-structured light [27]
Shading	Shading	Shape-from-shading [28]
		Photometric stereo [29]
Focus	Focus	Shape-from-focus [30]
		Shape-from-defocus [31]
Theodolite	Stereopsis	Trigonometry [32]

**Table 2:** Advantages and disadvantages of the most common sensor implementations, based on the basic principles for 3-D vision.

Basic Principle	Sensor/Technique	Advantages	Disadvantages
**Triangulation**	Consumer triangulation sensor (CTS)	-Off-the-shelf -Low cost -Provide RGB stream -Good community support, good documentation -Open source libraries available	-Vulnerable to sunlight, where no depth information is produced -Depth information is not possible at night or in very dark environments -Not weather resistant -Warm-up time required to stabilize the depth measurements (~1 h)
	Stereo vision	-Good community support, good documentation -Off-the-shelf smart cameras (with parallel computing) available -Robust enough for open field applications	-Low texture produce correspondence problems -Susceptible to direct sunlight -Computationally expensive -Depth range is highly dependent on the baseline distance
	Structure-from-motion	-Digital cameras are easily and economically available -Open source and commercial software for 3-D reconstruction -Suitable for aerial applications -Excellent portability	-Camera calibration and field references are a requirement for reliable measurements -Time consuming point cloud generation process is not suitable for real-time applications -Requires a lot of experience for obtaining good raw data
	Light sheet triangulation	-High precision -Fast image data acquisition and 3-D reconstruction -Limited working range due to the focus -Do not depend on external light sources -New versions have light filtering systems that allow them to handle sunlight	-High cost -Susceptible to sunlight -Time consuming data acquisition
**TOF**	TOF camera	-Active illumination independent of an external lighting source -Able to acquire data at night or in dark/low light conditions -Commercial 3-D sensors in agriculture are based on the fast-improving photonic mixer device (PMD) technology -New versions have pixel resolutions of up to 4.2 Megapixels -New versions have depth measurement ranges of up to 25 m	-Most of them have low pixel resolution -Most of them are susceptible to direct sunlight -High cost
	Light sheet (pulse modulated) LIDAR	-Emitted light beams and are robust against sunlight -Able to retrieve depth measurements at night or in dark environments -Robust against interference -Widely used in agricultural applications -Many research papers and information available -New versions perform well in adverse weather conditions (rain, snow, mist and dust)	-Poor performance in edge detection due the spacing between the light beams -Warm-up time required to stabilize the depth measurements (up to 2.5 h) -Normally bulky and with moving parts -Have problems under adverse weather conditions (rain, snow, mist and dust)
**Interferometry**	Optical coherent tomography (OCT)	-High accuracy -Near surface light penetration -High resolution	-High cost -Limited range -Highly-textured surfaces scatter the light beams -Relative measurements -Sensitive to vibrations -Difficult to implement

**Table 3:** Autonomous platforms for reducing the time-consuming and repetitive phenotyping practice.

Platform	Basic Principle	Shadowing Device	Environment	Institution	Type
Becam [73]	Triangulation	√	Open field	UMR-ITAP	Research
BoniRob [74]	TOF	√	Open field	Deepfield Robotics	Commercial
BredVision [75]	TOF	√	Open field	University of Applied Sciences Osnabrück	Research
Heliaphen [76]	Triangulation	×	Greenhouse	Optimalog	Research
Ladybird [77]	TOF and triangulation	√	Open field	University of Sidney	Research
Marvin [78]	Triangulation	√	Greenhouse	Wageningen University	Research
PhenoArch [79]	Triangulation	√	Greenhouse	INRA-LEPSE (by LemnaTec)	Research
Phenobot [80]	TOF and Triangulation	×	Greenhouse	Wageningen University	Research
PlantEye [81]	Triangulation	×	Greenhouse	Phenospex	Commercial
Robot gardener [82]	Triangulation	×	Indoor	GARNICS project	Research
SAS [83]	Triangulation	×	Greenhouse	Alci	Commercial
Scanalyzer [84]	Triangulation	√	Open field, Greenhouse	LemnaTec	Commercial
Spy-See [85]	TOF and Triangulation	×	Greenhouse	Wageningen University	Research
Zea [86]	Triangulation	√	Open field	Blue River	Commercial

**Table 4:** Summary of the technical difficulties of the 3-D techniques used in agricultural applications.

Basic Principle	Technique	Application	Technical Difficulties
**Triangulation**	Stereo vision	-Autonomous navigation [38,39,40,42,44,46] -Crop husbandry [71,98,100] -Animal husbandry [121,132]	-Blank pixels of some locations specially the ones that are further away from the camera -Low light (cloudy sky) affects 3-D point generation -Direct sunlight and shadows in a sunny day affect strongly the depth image generation -Uniform texture of long leaves affect the 3-D point generation -Limited field of view -External illumination is required for night implementations -Correspondence and parallax problems -A robust disparity estimation is difficult in areas of homogeneous colour or occlusion -Specular reflections -Colour heterogeneity of the target object -A constant altitude needs to be maintained if a stereo vision system is mounted on a UAV -Camera calibration is necessary -Occlusion of leaves -Selection of a suitable camera position
	Multi-view stereo	-Crop husbandry [65] -Animal husbandry [124]	-Surface integration from multiple views is the main obstacle -Challenging software engineering if high-resolution surface reconstruction is desired -Software obstacles associated with handling large images during system calibration and stereo matching
	Multiple-baseline stereo	-Autonomous navigation [43]	-Handling a rich 3-D data is computationally demanding
	Structure-from-motion	-Crop husbandry [64,67,68,69,97]	-Occlusion of leaves -Plant changing position from one image to the other due to the wind -High computation power is required to generate a dense point cloud -Determination of a suitable Image overlapping percentage -Greater hectare coverage requires higher altitudes when using UAVs -The camera’s pixel resolution determines the field spatial resolution -Image mosaicking is technically difficult from UAVs due to the translational and rotational movements of the camera
	Shape-from-Silhouette	-Crop husbandry [87,88,89]	-3-D reconstruction results strongly depend on good image pre-processing -Camera calibration is important if several cameras are used -Dense and random canopy branching is more difficult to reconstruct -Post-processing filtering may be required to remove noisy regions
	Structured light (light volume) sequentially coded	-Crop husbandry [93]	-Limited projector depth of field -High dynamic range scene -Internal reflections -Thin objects -Occlusions
	Structured light (light volume) pseudo random pattern	-Autonomous navigation [47] -Animal husbandry [122,128]	-Strong sensitivity to natural light -Small field of view -Smooth and shiny surfaces do not produce reliable depth measurements -Misalignment between the RGB and depth image due to the difference in pixel resolution -Time delay (30 s) for a stable depth measurement after a quick rotation -Mismatch between the RGB and depth images’ field of view and point of view
	Shape-from-Shading	-Crop husbandry [101]	-A zigzag effect at the target object’s boundary is generated (in interlaced video) if it moves at high speeds
	Structured light shadow Moiré	-Crop husbandry [94]	-Sensitive to disturbances (e.g., surface reflectivity) that become a source of noise
	Shape-from-focus	-Crop husbandry [90]	-Limited depth of field decreases the accuracy of the 3-D reconstruction
**TOF**	Pulse modulation (light sheet)	-Autonomous navigation [49] -Crop husbandry [106,109]	-Limited perception of the surrounding structures -Requires movement to obtain 3-D data -Pitching, rolling or jawing using servo motors (*i.e.*, pan-tilt unit) is a method to extend the field of view, but adds technical difficulties -Point cloud registration requires sensor fusion -Small plants are difficult to detect -Lower sampling rate and accuracy compared to continuous wave modulation TOF
	Pulse modulation (light volume)	-Autonomous navigation and crop husbandry [107]	-Limited pixel resolution -Difficulty to distinguish small structures with complex shapes
	Continuous wave modulation (light sheet)	-Crop husbandry [109]	-Poor distance range measurement (up to 3 m)
	Continuous wave modulation (light volume)	-Crop husbandry [110,111,112] -Animal husbandry [122]	-Small field of view -Low pixel resolution -Calibration could be required to correct radial distortion -Requires a sunlight cover for better results -Limited visibility due to occlusion -Lack of colour output that could be useful for a better image segmentation
**Inter-ferometry**	White-light	-Crop husbandry [115,116,120]	-The scattering surface of the plant forms speckles that affect the accuracy -Complexity of implementation
	Holographic	-Crop husbandry [119]	-Need of a reference object in the image to detect disturbances
	Speckle	-Crop husbandry [117,118]	-Agricultural products with rough surface could be difficult to reconstruct -High camera resolutions provide better capabilities to resolve high fringe densities
