# Correction: Vázquez-Arellano, M., *et al*. 3-D Imaging Systems for Agricultural Applications—A Review. *Sensors* 2016, *16*, 618

**DOI:** 10.3390/s16071039

**Published:** 2016-07-05

**Authors:** Manuel Vázquez-Arellano, Hans W. Griepentrog, David Reiser, Dimitris S. Paraforos

**Affiliations:** Institute of Agricultural Engineering, University of Hohenheim, Garbenstrasse 9, Stuttgart 70599, Germany; hw.griepentrog@uni-hohenheim.de (H.W.G.); dreiser@uni-hohenheim.de (D.R.); d.paraforos@uni-hohenheim.de (D.S.P.)

The authors wish to make the following corrections to Table 3 of the title paper [1]: the working environment of the PlantEye platform should be changed from “Greenhouse” to “Open field, Greenhouse” and the shadowing device of the Scanalyzer platform should be changed from *“√”* to *“×”*. The full corrected table is given below.

## Figures and Tables

**Table 3:** Autonomous platforms for reducing the time-consuming and repetitive phenotyping practice.

Platform	Basic Principle	Shadowing Device	Environment	Institution	Type
Becam [73]	Triangulation	√	Open field	UMR-ITAP	Research
BoniRob [74]	TOF	√	Open field	Deepfield Robotics	Commercial
BredVision [75]	TOF	√	Open field	University of Applied Sciences Osnabrück	Research
Heliaphen [76]	Triangulation	×	Greenhouse	Optimalog	Research
Ladybird [77]	TOF and Triangulation	√	Open field	University of Sidney	Research
Marvin [78]	Triangulation	√	Greenhouse	Wageningen University	Research
PhenoArch [79]	Triangulation	√	Greenhouse	INRA-LEPSE (by LemnaTec)	Research
Phenobot [80]	TOF and Triangulation	×	Greenhouse	Wageningen University	Research
PlantEye [81]	Triangulation	×	Open field, Greenhouse	Phenospex	Commercial
Robot gardener [82]	Triangulation	×	Indoor	GARNICS project	Research
SAS [83]	Triangulation	×	Greenhouse	Alci	Commercial
Scanalyzer [84]	Triangulation	×	Open field, Greenhouse	LemnaTec	Commercial
Spy-See [85]	TOF and Triangulation	×	Greenhouse	Wageningen University	Research
Zea [86]	Triangulation	√	Open field	Blue River	Commercial
